# Associations between physical activity levels and renal recovery following acute kidney injury stage 3: a feasibility study

**DOI:** 10.1186/s12882-022-02759-x

**Published:** 2022-04-11

**Authors:** Anam Asad, Amal Thomas, Maurice Dungey, Katherine L. Hull, Daniel S. March, James O. Burton

**Affiliations:** 1 Department of Cardiovascular Sciences, University of Leicester, Leicester, UK; 2 John Walls Renal Unit, Leicester General Hospital, Leicester, UK; 3 York Trials Unit, Department of Health Sciences, University of York, York, UK; 4 NIHR Leicester Biomedical Research Centre, Leicester, UK; 5 School of Sport, Exercise and Health Sciences, Loughborough University, Loughborough, UK

**Keywords:** Acute kidney injury, Physical activity, Renal recovery

## Abstract

**Background:**

Acute kidney injury (AKI) can lead to chronic kidney disease, which results in poor long-term outcomes. There is plausibility that increased levels of physical activity may promote renal recovery post-AKI. This study aimed to investigate associations between physical activity levels and renal recovery following stage 3 AKI, and to assess the feasibility of measuring physical activity levels in this population.

**Methods:**

Forty One hospitalised patients with AKI stage 3 were enrolled. Serum creatinine and estimated glomerular filtration rate (eGFR) were collected at 12 months prior to the development of AKI, during the hospital admission when the episode of AKI stage 3 occurred, and at 1-, 3- and 6-months post discharge. All participants completed the General Practice Activity Questionnaire (GPPAQ) to assess physical activity levels. A pedometer was also worn for 7 days immediately following discharge and at 6-months post discharge to ascertain an average daily step count. Feasibility outcomes including eligibility, recruitment and retention rates, and losses to follow up were also assessed.

**Results:**

The average (± SD) baseline eGFR and median (IQR) serum creatinine was 71 ± 20 mL/min/1.73m^2^ and 85 (49) μmol/L respectively. A threefold increase in creatinine occurred during hospitalisation 436 (265) μmol/L. Greatest renal recovery occurred prior to discharge, with recovery continuing for a further three months. Inactive individuals (low GPPAQ scores) had consistently higher serum creatinine values compared to those who were active: 1 months 122 (111) μmol/L vs 70 (0) μmol/L, 6 months 112 (57) μmol/L vs 68 (0) μmol/L. Individuals with higher step counts also displayed better renal recovery 6-months post discharge (*r* = -0.600, *p* = 0.208).

**Conclusions:**

Higher levels of physical activity are associated with improved renal recovery after 6- months following an episode of stage 3 AKI. A future randomised controlled trial is feasible and would be required to confirm these initial findings.

## Background

Patients with acute kidney injury (AKI) experience both short- and long-term adverse health outcomes [1]. The immediate complications such as electrolyte, fluid and acid–base disturbances, are well recognised and have established management strategies [2]. In contrast, the long-term effects of AKI that are conferred through the development of chronic kidney disease (CKD), such as increased risk of cardiovascular disease, mortality and progression to end-stage kidney disease (ESKD) are not addressed to the same extent [1]. It was previously thought that kidney damage completely resolves after AKI, however it is now understood that there is often sustained renal dysfunction [3]. Despite this, there are currently no targeted treatments aimed at improving post-AKI renal function. Many novel therapeutic interventions such as alpha melanocyte stimulated hormone, low dose furosemide and alkaline phosphatase have been trialled [4–6] with limited success, emphasising the requirement for new treatment strategies.

Data from animal models [7–13] demonstrate that increased levels of physical activity may be protective and promote renal recovery following an episode of chemically-induced AKI. The proposed mechanism includes the anti-inflammatory effects of increased activity [9, 10] attenuating renal damage through controlling low-grade chronic inflammation that may sustain renal damage. A reduction in fibrotic [8] and oxidative pathways, [8, 10, 11, 13] and autophagy upregulation [7, 11] improve cell survival and kidney function in physically active animals post AKI. These data suggest a possible role of exercise in attenuating renal damage post AKI, thereby improving patient outcomes.

Despite these promising animal models, at present there is a paucity of data on the association between exercise and physical activity levels and AKI recovery in humans. More specifically, since an increase in AKI severity is associated with an increased risk of CKD and ESKD [14], AKI stage 3 individuals would benefit the most from an improvement in their renal function post AKI. Additionally, as patients with AKI are usually acutely unwell and no previous study has tried to enrol these participants into a physical activity study, the feasibility of conducting such a study in this patient group is unclear.

Therefore, the aims of this observational study are to: explore the associations between physical activity levels and renal recovery following an episode of AKI stage 3 and; evaluate the feasibility of a study of physical activity in the AKI population.

## Methods

### Study design and population

This was an observational cohort study performed at the University Hospitals of Leicester (UHL) NHS Trust in the United Kingdom. The study received ethical approval by the UK National Health Service Research Ethics Committee (WALES REC 6 ref: 18/WA/0358), and all participants provided written informed consent.

Patients were identified through an electronic AKI alert system for hospitalised patients. Inclusion criteria: aged > 18; hospitalised with AKI stage 3; measurement of renal function available within the previous 12-month (baseline) and; willing and able to provide informed consent. AKI stage 3 was defined according to the Kidney Disease Improving Global Outcomes (KDIGO) guidelines [15]: an increase in serum creatinine equal to 3 times baseline levels or < 0.3 mL/kg/h urine output for ≥ 24 h. Baseline renal function was determined by a creatinine and eGFR measurement taken within 12 months prior to their admission. Exclusion criteria: AKI of obstructive aetiology, a previous AKI episode within the last month, previous solid organ transplant, baseline eGFR < 30 ml/min/ 1.73 m2, complete renal recovery or ongoing requirement for renal replacement therapy at time of discharge.

### Data collection

#### Physical activity

All enrolled participants completed the General Practice Physical Activity Questionnaire (GPPAQ) [16]. The GPPAQ measures current physical activity by providing a 4-level Physical Activity Index (PAI), categorising individuals into: inactive; moderately inactive; moderately active and active. All questionnaires were completed independently by the participant during their in-patient stay and all questions answered according to their abilities immediately prior to their hospital admission, to ascertain a baseline value. These questionnaires were repeated at 3- and 6-months post discharge.

All participants were given the option of wearing a YAMAX Digi-Walker SW800-SW801 (Yamax; Yamax Corporation, Tokyo, Japan) pedometer for 7 consecutive days and an average daily step count was calculated by dividing the total number of steps by days worn. Step count data were collected twice during the study: immediately after discharge and 6 months post discharge.

#### Renal function

Baseline participant characteristics and measures of renal function were extracted from medical records (Integrated Clinical Environment (ICE), Sunquest Information Systems, Tucson, Arizona) during the hospital admission and at time of discharge. Creatinine was measured again at 1-, 3- and 6-months post discharge.

#### Feasibility outcomes

The feasibility outcomes included: the number of eligible patients against the number screened; the proportion of eligible patients enrolling in the study against those that are eligible and assessment of the proportion of participants followed up to 6-months with complete outcome data.

#### Participant number and sample size

As a feasibility study, a power calculation to determine a sample size was not performed. In line with previous data for feasibility trials [17], approximately 40 participants are adequate to address the proposed feasibility outcomes.

### Statistical analysis

All data were assessed for normality and are presented as mean ± standard deviation (SD) or median ± interquartile ranges (IQR) if skewed. Categorical variables are reported as a frequency and percentage. Renal recovery was assessed by calculating the absolute differences between follow up (1- and 6-month) and baseline creatinine values. A percentage difference was also calculated by dividing the calculated absolute difference by the baseline creatinine value. There is no consensus definition of renal recovery following AKI with recovery definitions ranging between a return of serum creatinine within 10–50% [18–21] of baseline values. In line with the published literature, we defined ‘good’ renal recovery as serum creatinine returning to within 25% of the baseline value [22–24].

## Results

### Patient demographics

Forty One participants were recruited from March 2019 to December 2020. Complete questionnaire data were available from 20 patients at 6 months. All participant baseline demographic data are presented in Table 1.

**Table 1:** Baseline Patient Demographics

Characteristic	All *N* = 41
**Age (years)**	
Mean (SD)	68 (± 17)
**Sex N (%)**	
Male	26 (63)
Female	15 (37)
**Ethnicity N (%)**	
White	40 (98)
Black	1 (2)
**Comorbidities N (%)**	
CKD	11 (28)
Hypertension	20 (49)
Diabetes Mellitus	11 (28)
Dyslipidaemia	7 (18)
Heart Failure	3 (8)
Malignancy	6 (15)
**Cause of AKI N (%)**	
Sepsis	20 (49)
Dehydration	13 (32)
Drug Induced	1 (2)
Chemotherapy Induced	2 (5)
Contrast Induced	1 (2)
Post-Operative	2 (5)
Rhabdomyolysis	1 (2)
Unknown	1 (2)
**Length of Admission (days)**	
Mean (SD)	15 (± 10)
**RRT Required**	
Yes N (%)	4 (10)
No N (%)	37 (90)
**ITU Admission**	
Yes N (%)	8 (20)
Length of ITU Admission (days)	7 (± 6)
No N (%)	33 (80)

*SD* Standard deviation, *CKD* Chronic kidney disease, *AKI* Acute kidney injury, *ITU* Intensive treatment unit, *RRT* Renal replacement therapy

### Feasibility outcomes

**Fig. 1:**
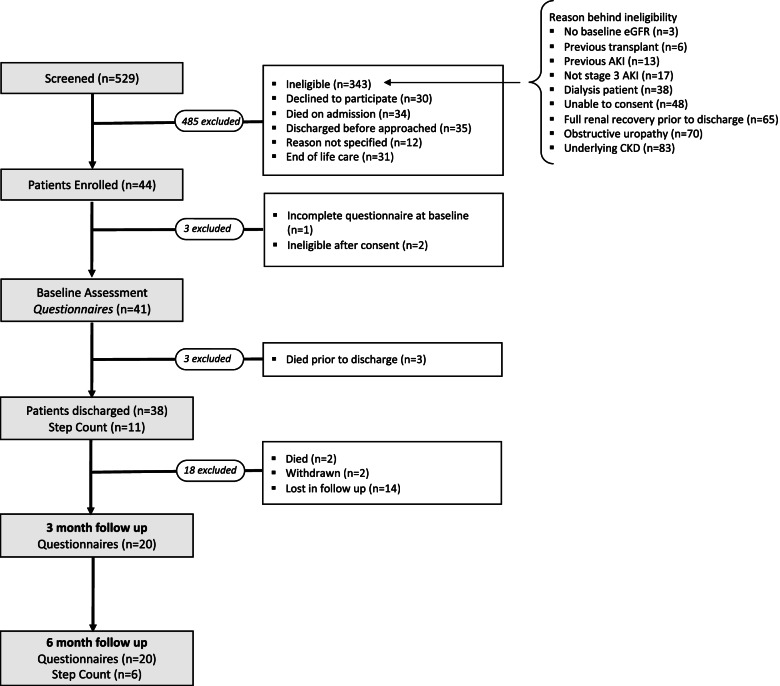
Flow chart of patient recruitment. AKI = acute kidney injury; CKD = chronic kidney disease; eGFR = estimated glomerular filtration rate

Five hundred twenty-nine patients were screened for eligibility, with 186 (35%) individuals being eligible for enrolment (see Fig. 1). 74 (14%) patients were approached, with 44 (8%) providing informed consent to participate. 41 participants completed all baseline assessments out of which 11 (27%) also consented to monitoring their daily step count for 7 days after discharge. 38 participants were successfully discharged from hospital (3 participants died before discharge), and all were contacted to complete the questionnaires at 3- and 6-months post discharge. During follow up, 2 individuals chose to withdraw from the study and 2 participants died. In total, 20 (52%) participants completed follow up questionnaire data with measures of renal function being available for 12 and 13 participants at 3 and 6 months respectively (Fig. 1).

### Renal function

**Fig. 2:**
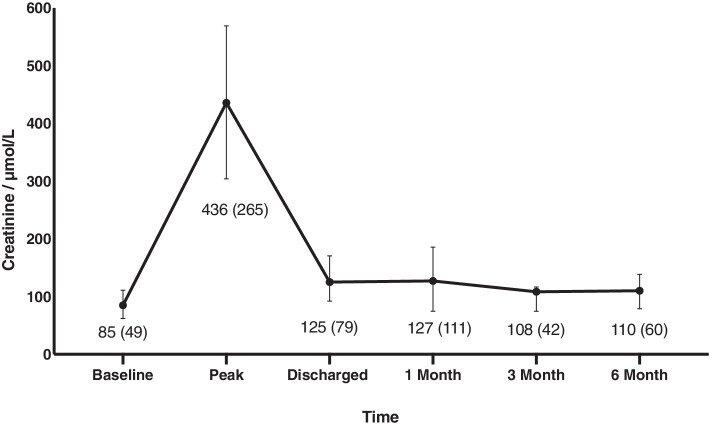
Renal Function throughout the study. Renal function estimated by creatinine is reported as median (IQR) for all participants available at each time point—baseline, discharge and 1,3- and 6-months post discharge. The error bars demonstrate IQR

All participants experienced AKI stage 3, shown through a minimum threefold increase (from 85 (49) to 436 (265) μmol/L in serum creatinine levels during hospitalisation (Fig. 2). At discharge, residual renal impairment persisted, 125 (79) μmol/L. Renal recovery continued at 3-months post discharge, 108 (42) μmol/L, but with no further recovery being noted at 6-months, 110 (60) μmol/L (Fig. 2).

### Physical activity levels

#### GPPAQ

**Fig. 3:**
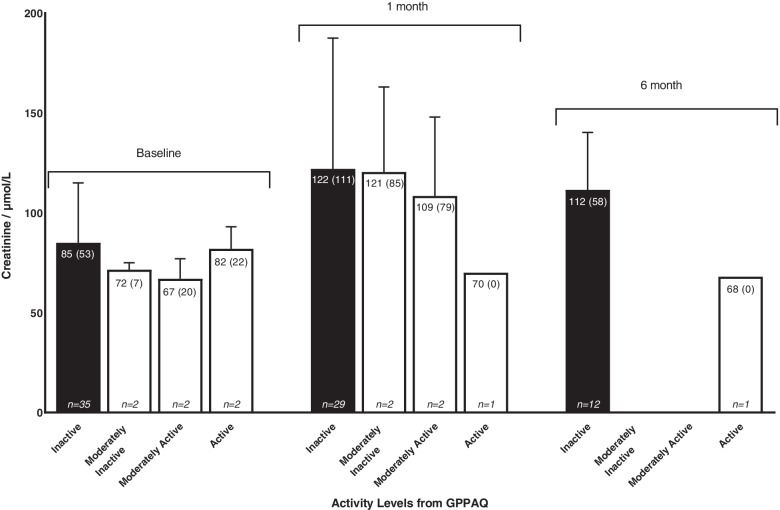
Creatinine values for each activity level from the GPPAQ shown at baseline and 1 and 6 months post discharge. Median (IQR) creatinine values presented with the number of participants at each activity levels displayed at the bottom of each bar (*n* =)

The majority of participants recorded high levels of inactivity (Fig. 3). At baseline, moderately active individuals had a lower median (IQR) creatinine compared to those who were inactive, 67 (10) μmol/L vs 85 (49) μmol/L. This pattern, showing active individuals having lower creatinine values and hence better renal function, is repeated at each time point during the study, Fig. 3.

#### Renal function and step count data

**Fig. 4:**
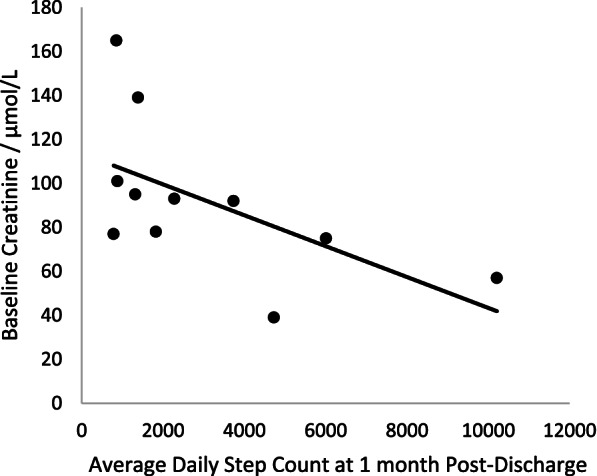
Correlation between 1-month average step count and baseline renal function (*n* = 11), Spearman’s rho = -0.6636, *p* = 0.03. Both continuous variables were assessed for normality. Spearman’s rank test was used to assess correlation. A significant negative association was found between creatinine value taken 12 months prior to AKI and average step count immediately following hospital discharge

A higher baseline renal function (lower creatinine level), taken 12-months prior to AKI, was associated with a higher step count immediately after discharge, (*r* = -0.6636, *p* = 0.03), Fig. 4. At 1-month, those who had recovered to within 25% of their baseline creatinine had a higher average daily step count compared to those who were further away from their baseline 3712 (± 3960) vs 3085 (± 2202). Individuals with higher step counts at 6-months had better renal recovery at the same time point, (*r* = -0.600, *p* = 0.2080) Fig. 5.

**Fig. 5:**
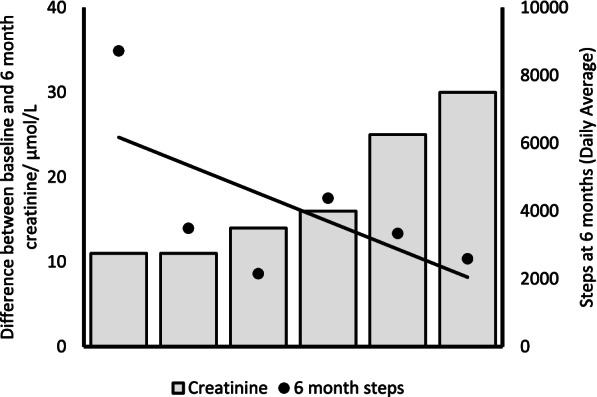
Correlation between renal recovery and average daily step count at 6 months. Spearman’s rho = -0.600, *p* = 0.21. Each bar on the graph represents a participant’s renal recovery (ie the absolute difference between their creatinine at 6 months post discharge and their baseline creatinine, therefore a greater difference indicates worse renal recovery). Each data point (dot) shown on the graph is their average daily step count. The line shows the association between the renal recovery and the average daily step count

## Discussion

This is the first study to measure physical activity in individuals following an episode of stage 3 AKI. We observed an association between higher levels of physical activity and improved renal function. Individuals with higher physical activity levels, assessed via the GPPAQ questionnaire, had consistently lower creatinine levels at 1- and 6-months post discharge. This association is in keeping with data from the 6-month step count as those with a higher average daily step count had greater renal recovery at 6 months post-discharge. We also observed an association between higher levels of physical activity prior to AKI and better recovery in the post AKI period. We observed in patients with AKI stage 3 that renal impairment persisted at discharge with maximum renal recovery being achieved 3 months post discharge, as no further recovery was noted at 6 months. Lastly, we measured the feasibility of a study observing physical activity levels in individuals following an episode of AKI. We screened a large number of individuals with only few meeting the eligibility criteria and being subsequently enrolled to participate. Additionally, many participants were lost to follow up at 3 and 6 months.

Numerous pre-clinical animal studies show that increasing physical activity attenuates renal damage post-AKI, highlighting the fact that there is biologically plausibility behind our findings. The transferability of these animal findings in the human setting has been questioned [25, 26]; however our results support the association between improved renal recovery in more physically active individuals [27]. Physical activity in the AKI population has not previously been reported. Furthermore, we found no studies which correlate physical activity with renal recovery in the AKI setting. However, the pattern of renal recovery observed in our study, whereby recovery continued post discharge, is well established [28]. Furthermore, it is known that those with recovery following AKI have better outcomes than those with persistent renal impairment. In addition, time to recovery is a valuable clinical parameter as those with longer recovery times have worse long-term outcomes such as a higher incidence of CKD, long term dialysis use and increase in mortality [29]. This study observed that the greatest amount of renal recovery occurred in the 3 months post-discharge, therefore any future interventions aimed at recovering renal function would be most beneficial if implemented during this time frame.

The association observed between increased levels of physical activity and improved renal recovery does not imply causation. It is possible that individuals may have been more active because of improved renal recovery. Furthermore, those who were more active prior to AKI may have had better overall health hence facilitating their faster recovery in renal function. This is supported by studies [30, 31] that report associations between poor pre-operative cardiorespiratory functioning and worse post-operative outcomes, such as longer stay in intensive care and increased short- and long-term morbidity and mortality. Additionally, randomised controlled trial (RCT) data has demonstrated that pre-operative exercise training decreases post-operative complications in patients undergoing elective abdominal aortic aneurysm repair [32] and coronary artery bypass graft surgery [33], indicating that healthier individuals prior to hospitalisation have better outcomes. It is therefore not clear whether our observations showing associations between improved recovery and renal function post AKI have implications for long-term outcomes in this population. Furthermore, it is unclear whether increasing physical activity levels (through a targeted intervention) following AKI, would lead to an improvement in renal recovery. Regardless, since our study demonstrates an association between increased levels of physical activity and improved renal function, this warrants further investigation.

In this study only a small number of the screened patients fulfilled our inclusion criteria. The three most common reasons for ineligibility were: underlying severe CKD, obstructive uropathy and patients achieving full renal recovery prior to discharge. A wider inclusion criteria should be considered for future studies. This can be achieved by including individuals with stage 1 and stage 2 AKI. More specifically, those with an increased risk of CKD progression following AKI should be identified and recruited. Whilst increase AKI severity is a risk factor for CKD progression, other factors such as the number of AKI insults and presence of other CKD risk factors such as diabetes mellitus and hypertension are also important to consider [34].

Out of those who were approached, the majority (59%) consented to participate. We found large losses to follow up at both 3- and 6-months post discharge. Only a small amount of these were attributed to participant death and withdrawal of consent from the study. Based on prior research, it is possible that difficulties in participant retention may be explained by our cohort belonging to an older age demographic, 68 (± 17) years. Difficulties in both recruitment and retention in > 65 years have been previously demonstrated [35]. Decline in overall health, loss of interest, failure to recognise research benefit and increase frailty have previously been reported as reasons for lack of retention in research studies in this age group [35, 36]. It is plausible that our study could also have been impacted by these factors. Different strategies have been proposed on how to combat these issues. Many of which were already part of our study design, most notably the remote nature of our data collection process whereby individuals were able to complete the follow up questionnaires at home. Methods to increase retention in future studies include regular follow up phone calls to keep participants engaged in the study. Widening the inclusion criteria to the possible addition of stage 1 and stage 2 AKI individuals would also create a more age-diverse cohort hence addressing the issues seen mostly in > 65 year demographic. With the successful addition of strategies to reduce loss of follow up, our data supports that larger scale studies of physical activity would be feasible in the AKI population.

These data demonstrate that a RCT designed to investigate the effect of exercise on renal recovery post AKI is feasible. At present, there are still no effective interventions that improve renal recovery and thus reduces the long term sequalae of an episode of AKI [37]. This feasibility study provides hypothesis generating data, however due to the limitations of our sample size, the results must be interpreted with caution. A future, large scale RCT is needed to definitively test the hypothesis that a programme of exercise may have a positive impact upon renal recovery.

### Limitations

A small sample size and a large proportion of patients lost to follow are the primary limitations of this study, although the design was to test feasibility rather than to show a definitive result. Loss of follow up during the study occurred due to withdrawal, patient death and lack of response to the follow-up questionnaire, the reasons behind which were not explored. Selection and recruitment bias may have resulted in the recruitment of more active individuals, limiting the generalisability of the findings. Whilst we observed an association between increased levels of physical activity and improved renal recovery, due to the observational study design, a causal relationship cannot be inferred.

## Conclusion

This feasibility study demonstrates a possible association between higher levels of physical activity and improved renal recovery after 3-months following an episode of stage 3 AKI. A future randomised controlled trial is feasible and would be required to confirm these initial findings.

## Data Availability

The datasets generated and/or analysed during the current study are not publicly available. Consent and approval were given for secure storage of data within the University of Leicester and University of Leicester Hospitals Trust servers only. As such, anonymised data will be available on request once details of secure transfer and storage are clarified with those research teams.

## Abbreviations

AKI: Acute kidney injury
CKD: Chronic kidney disease
ESKD: End stage kidney disease
UHL: University Hospitals Leicester
NHS: National health service
KDIGO: Kidney Disease Improving Global Outcomes
GFR: Glomerular filtration rate
GPPAQ: General Practice Physical Activity Questionnaire
PAI: Physical Activity Index
ICE: Integrated Clinical Environment
SD: Standard deviation
IQR: Interquartile range
RCT: Randomised controlled trial
ITU: Intensive care unit
